# SUBJECTIVE APPRAISALS OF PHYSICAL AND EMOTIONAL HEALTH: EXAMINING AGE DIFFERENCES ACROSS RURAL URBAN COMMUNITIES

**DOI:** 10.1093/geroni/igz038.1179

**Published:** 2019-11-08

**Authors:** Hillary Dorman, Rebecca S Allen, Patricia A Parmelee, Lisa C McGuire

**Affiliations:** 1 University of Alabama, Tuscaloosa, Tuscaloosa, Alabama, United States; 2 The University of Alabama, Tuscaloosa, Alabama, United States; 3 CDC, Atlanta, Georgia, United States

## Abstract

Using the Meikirch model as a theoretical underpinning, the present study aimed to examine population health disparities by discerning age variations within and across rural urban areas. Secondary data analysis was conducted using the CDC’s 2017 Behavioral Risk Factor Surveillance System. The study’s outcome variables included physical health and mental health burden (zero days, 1-13 days, 14+ days). A total sample of 96,568 adults were included with a mean age of 66.05 years (SD = 9.91). Individuals were classified in the following age groups: 43% middle-aged (45-64 years), 33% young-old (65-74 years), and 25% old-old adults (75+ years). The sample was largely female (61%), Non-Hispanic White (86%), and urban (67%). A series of chi-square tests of independence – post hoc tests when applicable – were completed. Overall, rural residents reported a higher prevalence of severe physical and mental health symptom burden. Regarding physical health burden, a significant difference was found within urban settings (X2(4) = 50.74, p < .001), where, unexpectedly, young-old adults reported the best physical health. Regarding mental health burden, a significant difference was found for both urban (X2(4) = 1661.72, p < .001)) and rural settings (X2(4) = 820.65, p < .001), with middle-aged adults reporting greater mental illness and the old-old adults reporting greater mental health resiliency. Findings suggest that a multidimensional framework of health usefully informs public health and clinical service interventions, identifying populations and locations in need (i.e., targeting rural physical health across age groups and mental health among the middle-age, regardless of location).

